# Supplementary material for: The interactome of intact mitochondria by cross-linking mass spectrometry provides evidence for coexisting respiratory supercomplexes
Source: Mol Cell Proteomics. 2017 Dec 8;17(2):216–32. doi: 10.1074/mcp.RA117.000470 (PMC5795388; doi:10.1074/mcp.RA117.000470)
Supplement: Supplemental Data [file supp_RA117.000470_133922_0_supp_23978_fzffwf.zip › spectra_annotation/mito_spectra_annotation/389-1-7-1-35-1.pdf]

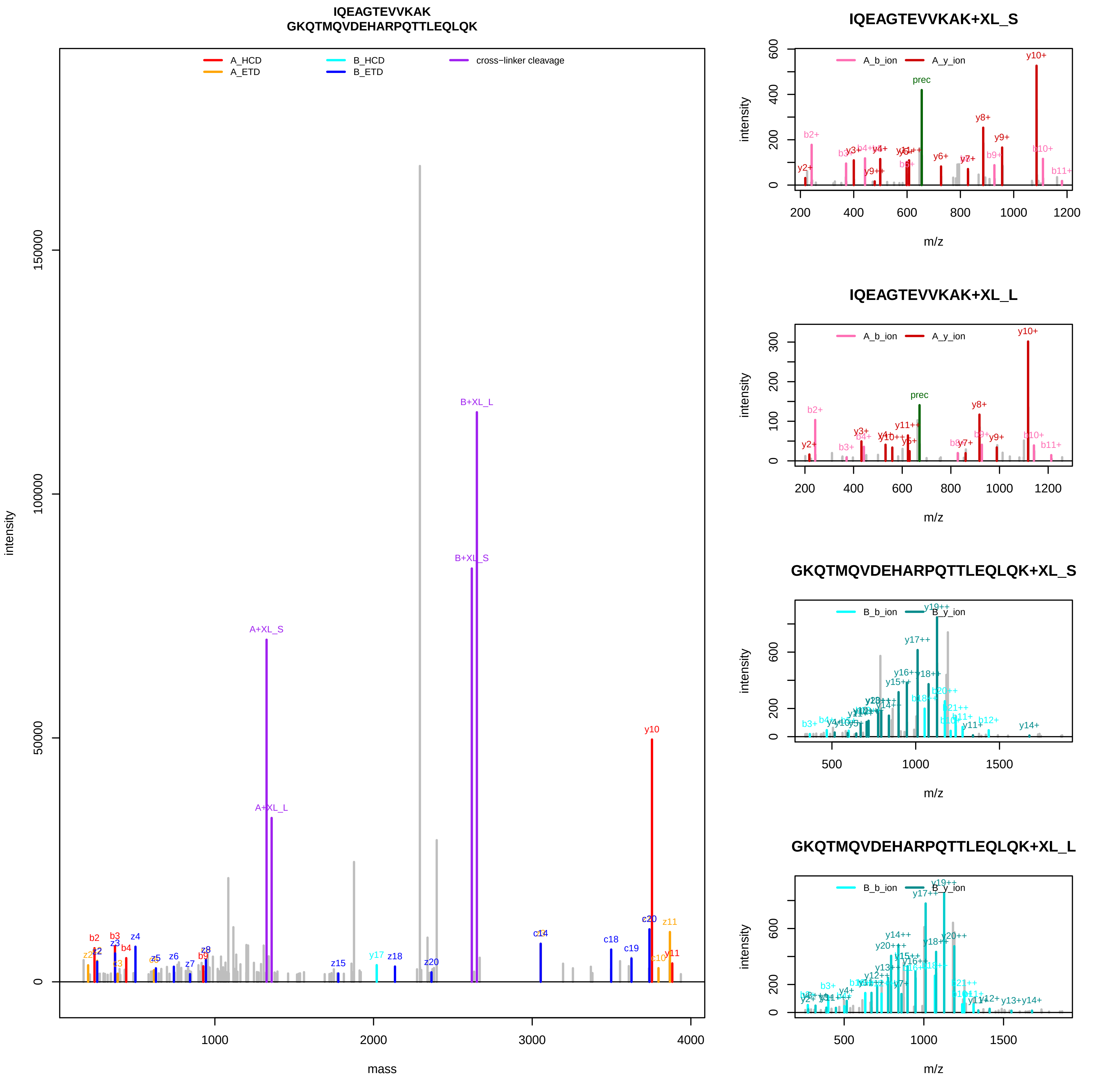

IQEAGTEVVKAK
GKQTMQVDEHARPQTTLEQLQK
A_HCD
A_ETD
B_HCD
B_ETD
cross−linker cleavage
intensity
mass
0
50000
100000
150000
1000
2000
3000
4000
A+XL_S
A+XL_L
B+XL_L
B+XL_S
y10
z11
c20
c18
c19
c14
z20
z18
y17
z15
b2
b3
b4
z4
z3
z2
z5
z6
z7
z8
b9
y11
c10
IQEAGTEVVKAK+XL_S
A_b_ion
A_y_ion
prec
y10+
y8+
y9+
b2+
b10+
b11+
y2+
y6+
m/z
IQEAGTEVVKAK+XL_L
y10+
prec
y8+
y9+
b2+
b10+
b11+
y2+
y3+
GKQTMQVDEHARPQTTLEQLQK+XL_S
B_b_ion
B_y_ion
y19++
y17++
y16++
y18++
y15++
b20++
b21++
b12++
y14+
y11+
b3+
500
1000
1500
GKQTMQVDEHARPQTTLEQLQK+XL_L
y19++
y17++
y14++
y20++
y18++
y20++
y15++
y16++
y13++
y12++
b21++
y12+
y13+
y14+
